# Infectious disease burden in Gujarat (2005–2011): comparison of selected infectious disease rates with India

**DOI:** 10.3402/ehtj.v7.22838

**Published:** 2014-03-19

**Authors:** Veena Iyer, Gulrez Shah Azhar, Nandini Choudhury, Vidwan Singh Dhruwey, Russell Dacombe, Ashish Upadhyay

**Affiliations:** 1 Indian Institute of Public Health, Gandhinagar, Ahmedabad, India; 2 Integrated Disease Surveillance Project, Commissionerate of Health, Medical Services, Medical Education and Research, Government of Gujarat, Gandhinagar, India; 3 Liverpool School of Tropical Medicine, Liverpool, UK

**Keywords:** communicable diseases, dengue, enteric fever, infectious disease surveillance, laboratory confirmed, National Health Profile, urban dominance, viral hepatitis

## Abstract

**Background:**

India is known to be endemic to numerous infectious diseases. The infectious disease profile of India is changing due to increased human environmental interactions, urbanisation and climate change. There are also predictions of explosive growth in infectious and zoonotic diseases. The Integrated Disease Surveillance Project (IDSP) was implemented in Gujarat in 2004.

**Methods:**

We analysed IDSP data on seven laboratory confirmed infectious diseases from 2005–2011 on temporal and spatial trends and compared this to the National Health Profile (NHP) data for the same period and with other literature. We chose laboratory cases data for Enteric fever, Cholera, Hepatitis, Dengue, Chikungunya, Measles and Diphtheria in the state since well designed vertical programs do not exist for these diseases. Statistical and GIS analysis was done using appropriate software.

**Results:**

Our analysis shows that the existing surveillance system in the state is predominantly reporting urban cases. There are wide variations among reported cases within the state with reports of Enteric fever and Measles being less than half of the national average, while Cholera, Viral Hepatitis and Dengue being nearly double.

**Conclusions:**

We found some limitations in the IDSP system with regard to the number of reporting units and cases in the background of a mixed health system with multiplicity of treatment providers and payment mechanisms. Despite these limitations, IDSP can be strengthened into a comprehensive surveillance system capable of tackling the challenge of reversing the endemicity of these diseases and preventing the emergence of others.

## Introduction

India is known to be endemic to a number of infectious diseases ranging from age-old scourges like cholera to relatively recent ones like dengue (1–3). In order to be able to address this burden effectively, a true estimate of the burden of infectious diseases is essential (4). In the ensuing article, we have analysed data from the Integrated Disease Surveillance Project (ISDP) to estimate the rates of select infectious diseases in the state of Gujarat and compared these to published data for the rest of India.

As per the 2011-census figures, the population of Gujarat is 60 million. It has increased by 9.7 million (19% decadal growth) over the last census figure. Interestingly this growth is not uniform; the urban growth rate at 35.8% is higher than its rural counterpart at 9.2%. The urban expansion is taking place in the form of unplanned and haphazard growth in the outskirts of existing towns and cities with the appearance of new urban clusters (5). Consequently, there are predictions of explosive growth in certain infectious and zoonotic diseases due to forced man–environment interactions (6). Climate change has also been predicted to alter the infectious disease profile of the country with the emergence of new infectious diseases (7).

The IDSP was set up in 2004 with assistance from the World Bank to improve information available to government health services and private health care providers on a set of high-priority diseases and risk factors and respond to them effectively and promptly (8–10). These core reportable conditions included common infectious diseases, risk factors for non-communicable diseases, air and water quality indicators and road traffic accidents. All 35 states in the country were to be networked in three phases for this ambitious surveillance plan. However, it became clear during the first phase that there were insurmountable technical, managerial and financial hurdles in the immediate term. This resulted in the project being scaled down to nine states including Gujarat in 2009 (10).

IDSP Gujarat has been collecting and reporting surveillance data on acute diarrheal disease, bacillary dysentery, viral hepatitis, enteric fever, malaria, dengue/DHF/DSS, chikungunya, acute encephalitis syndrome, meningitis, measles, diphtheria, pertussis, chicken pox, fever of unknown origin (PUO), acute respiratory infection (ARI)/influenza like illness (ILI), pneumonia, leptospirosis, acute flaccid paralysis, dog bite and snake bite since 2005. These diseases are reported to the IDSP through three mechanisms, namely syndromic, presumptive and laboratory confirmed, using S, P and L forms, respectively (11, 12). Of these, laboratory confirmed reports provide the most accurate picture for enteric fever, cholera, dengue, chikungunya, diphtheria, Japanese encephalitis, shigella, meningococcal meningitis, viral hepatitis, leptospirosis and malaria. For viral Hepatitis, IDSP reports cases based on a simple algorithm of clinically diagnosed and laboratory confirmed non-B Hepatitis. In the case of measles, all clinically diagnosed cases are reported, though only a few samples in an outbreak situation undergo laboratory investigations.

The National Health Profile (NHP) is an annual publication of the Central Bureau of Health Intelligence (CBHI), a national nodal institution for health intelligence in India (13). One segment of the NHP, called Health Status Indicators, lists cases and deaths for communicable and non-communicable diseases and reproductive health conditions, which it compiles from data submitted by each state. For infectious diseases, each state's National Vector Borne Diseases Control Programme (NVBDCP) and the Directorate of Health Services submit laboratory confirmed reports on important air- and water-borne diseases.

We decided to analyse data on seven laboratory confirmed infectious diseases, namely enteric fever, cholera, hepatitis, dengue, chikungunya, measles and diphtheria collected and reported by Gujarat IDSP since 2005 and compare these rates to that in the literature from other parts of India. The rationale for this choice was that well-designed vertical programmes and infrastructure (including dedicated laboratories) already exist for diseases such as HIV (14), TB (15), polio (16, 17) and malaria (18, 19, 20). However, there has not been a determined response to diseases such as enteric fever, cholera, hepatitis, dengue, chikungunya, measles and diphtheria.

## Materials and methods

We collated district-wise and disease-wise disaggregated data from Gujarat IDSP's Annual Reports (12) from 2005 to 2011 and unpublished IDSP surveillance data for the year 2008, when the annual report was not published. We calculated annual rates from the raw data and then analysed for epidemiological profile, including temporal and spatial trends, rural–urban distributions and annual case rates. The annual case rates were calculated by averaging the cases reported between 2005 and 2011 and reported the cases per 100,000 population. All case disease rates were calculated based on the 2011-census population. We also mapped these data at the level of districts and urban centres using ESRI–Arc-GIS software. We compared IDSP Gujarat's case rates with case rates from the national data by similarly analysing annually published figures in the NHP of the CBHI for the same period (13).

We validated these data through interviews of IDSP staff at the state level. We visited the IDSP reporting units at the district level to observe the disease reporting process. We met with Rapid Response Unit members who responded to these outbreaks. We also had discussions with the entomologist and the epidemiologists working for IDSP.

We conducted an extensive literature search in Medline and Google Scholar databases for scientific papers and reports for prevalence and incidence studies on our selected diseases. The search queries used both free text and medical subject heading terms for names of our selected disease and epidemiological terms (incidence, prevalence, case rates). The limits applied were that the work should have been in India and in the past decade. We excluded animal studies and case reports. We performed citation tracking of important papers to ensure that relevant (unindexed) papers were not missed in electronic searches. The case rates for the diseases in the literature were calculated from the cases reported in them in the same way as for IDSP and NHP data.

To ensure that data entry and analysis were free from bias, an expert who was unaware of the disease profile in the state performed data entry. This analysis was scrutinised for errors by duplication and again peer reviewed by other experts who were not part of this study. Ethical clearance for this project was obtained through the Institutional Review Board of Public Health Foundation of India, New Delhi, by blinded external reviewers.

## Results

A secondary analysis of the seven diseases, namely enteric fever, cholera, hepatitis (water-borne), dengue, chikungunya (vector-borne), measles and diphtheria (vaccine-preventable) reported by the state IDSP from 2005 to 2011 is depicted in Table 1. Enteric fever, cholera and diphtheria are bacterial while the rest are viral. While data for six of the diseases were available since 2005, data for chikungunya were available only for 2010–2011.

**Table 1:** Epidemiological profile of selected diseases in Gujarat reported by IDSP during a 7-year period (2005–2011)

				Temporal analysis	
				
Diseases reported by IDSP	Total reported cases 2005–2011	Average reported case rates/100,000 population/year	Urban proportion over 7 years (%)	Ratio of 2011:2005 cases	Trendline
Enteric fever	112,884	26.71	36.80	2.02	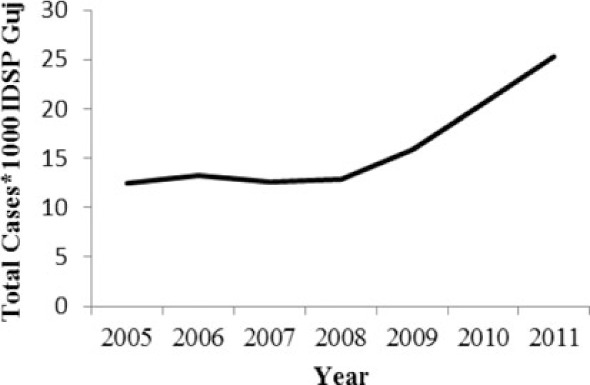
Hepatitis	104,722	24.76	51.07	2.20	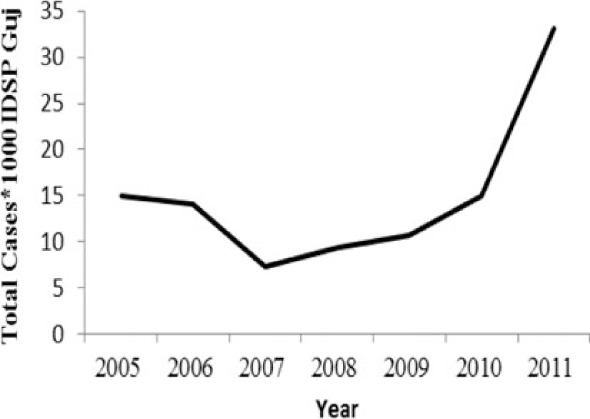
Cholera	1,560	0.40	83.42	1.80	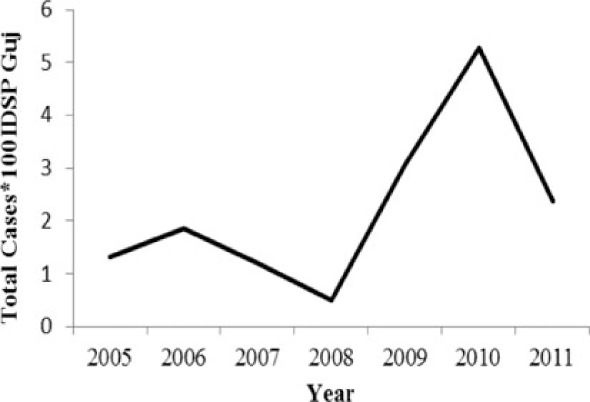
Dengue	10,403	2.46	75.56	2.15	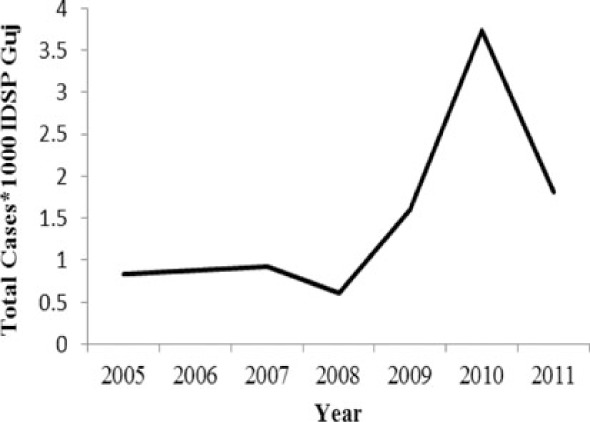
Chikungunya#	620	0.51	68.73	–	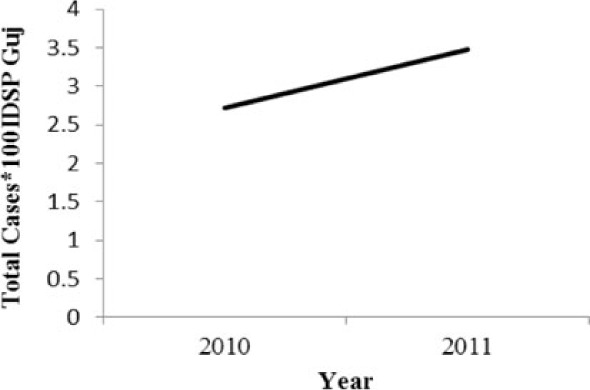
Measles	7,053	1.67	38.40	3.62	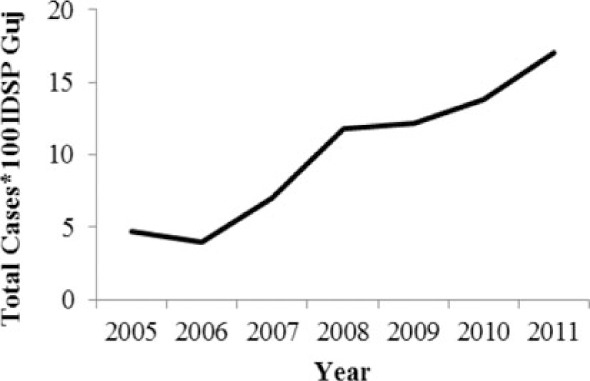
Diphtheria	1,461	0.35	89.12	0.60	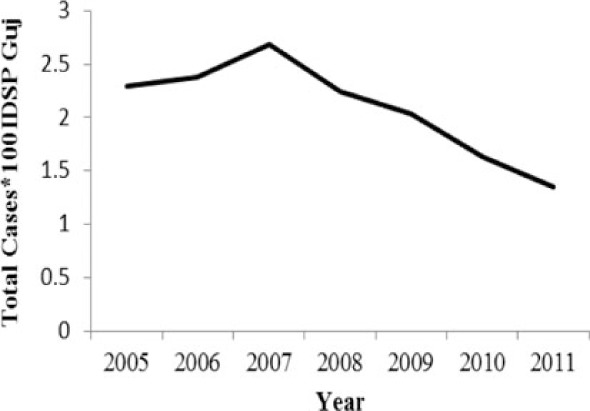

# Data available for 2010–2011.

During this 7-year period, for every 100,000 people in the state, approximately 186 have reportedly suffered from enteric fever, 173 from viral hepatitis, 17 from dengue, 11 from measles and 2 from diphtheria. The yearly average number of cases per 100,000 population is similar for enteric fever and hepatitis, 27 and 25, respectively.

Interestingly, more than 70% of the cases of chikungunya, dengue, cholera and diphtheria were reported by urban reporting units, that is, by the office of the Medical Officer for Health in seven municipal corporations in the state. The same for enteric fever and measles was only around 35%. Also, the two diseases, cholera and dengue showed an unusually sharp decline in the number of cases during 2011, although the reported cases of both these diseases seem to have doubled between 2005 and 2009. Only diphtheria showed a steady decline in reported cases from 2005 to 2011, reducing to half in 2011. Although data for chikungunya is available only for 2010 and 2011, it also shows an increase in the number of cases in 2011.

**Fig. 1:**
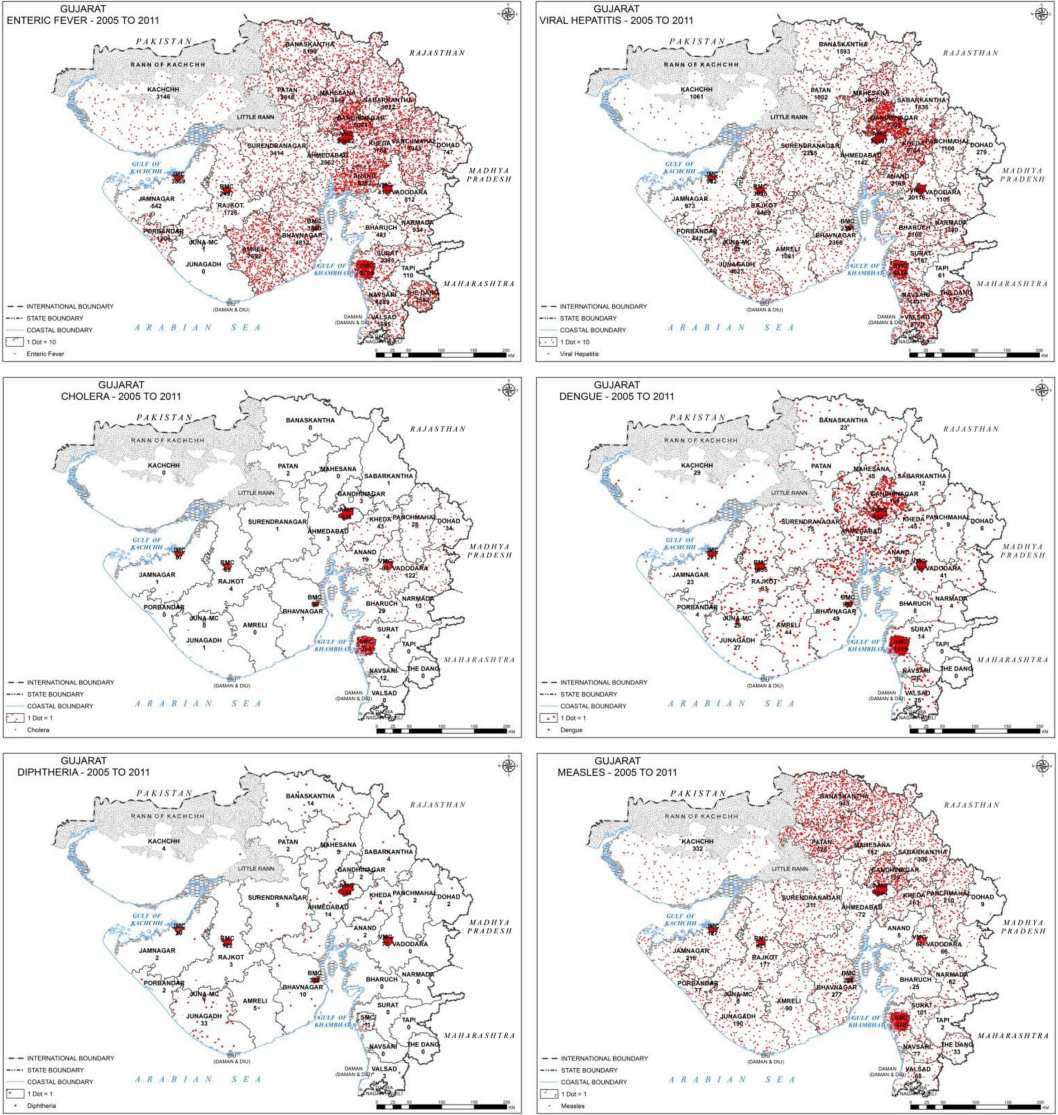
Distribution of cases of selected diseases by 25 districts and seven large urban centres from 2005 to 2011.

The spatial distribution of diseases in Fig. 1 also highlights the high urban proportion of all the diseases. Even though the distribution of enteric fever and measles is more rural than the other diseases, there is still a concentration of cases in urban areas. The map for enteric fever shows a preponderance of cases in Amreli district. The Dangs, a small tribal district reported a very large number of both enteric fever and hepatitis cases. Adjoining districts of Rajkot and Junagadh show a concentration of hepatitis cases, in contrast to the relatively low rate in their neighbouring districts (and even the state). Rural east Gujarat (especially Vadodara) shows a predominance of cholera cases. Dengue is prominent in central Gujarat (especially Ahmedabad and surrounding districts) and urban areas whereas remote tribal districts such as Tapi and The Dangs have not reported a single case. Diphtheria is also largely reported from urban areas, Junagadh district being the only non-urban area to have a higher case rate of Diphtheria.

**Fig. 2:**
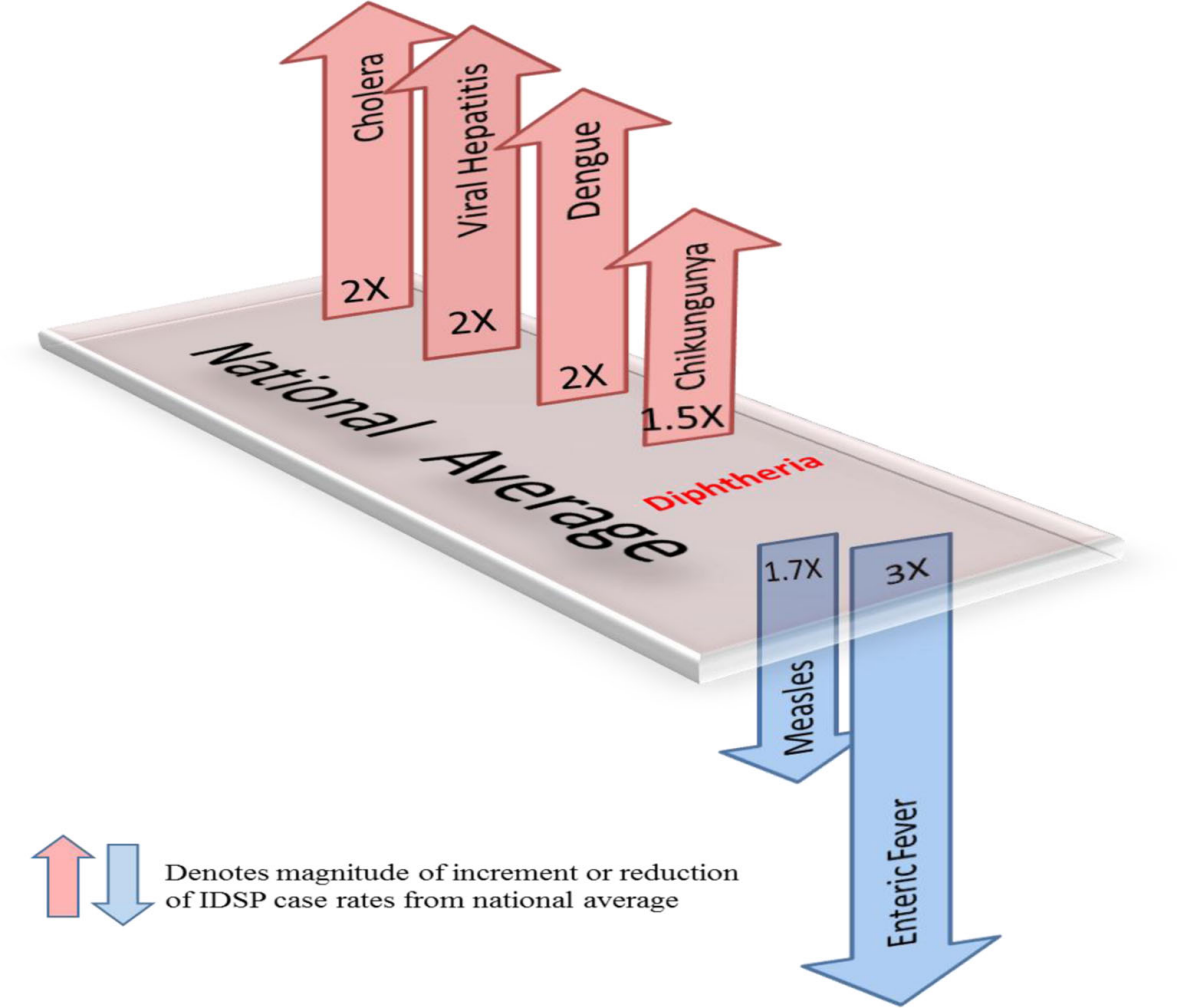
Measured against the national average reported by CBHI, Gujarat IDSP reported twice the case rates for cholera, viral hepatitis, dengue and chikungunya and two-third and one-third the rates of measles and enteric fever from 2005 to 2011.

**Fig. 3:**
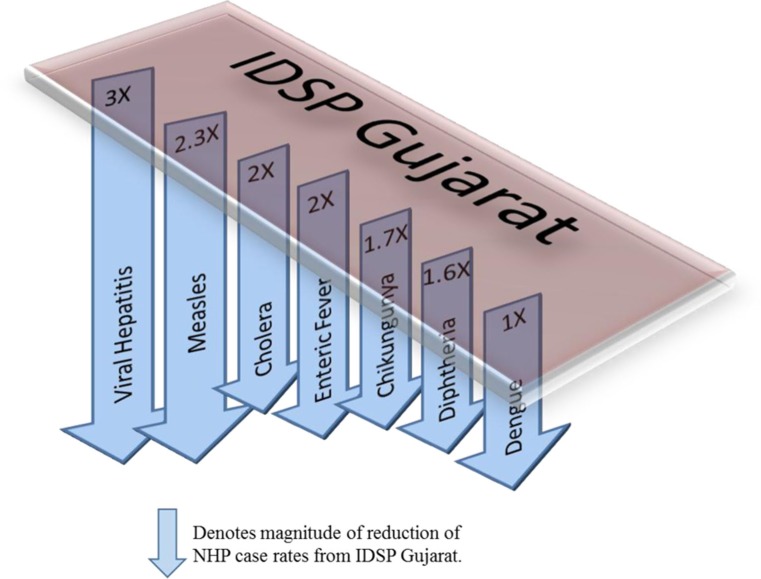
CBHI reports less than half of the case rates for all the diseases compared to those reported by IDSP Gujarat from 2005 to 2011.

Table 2 and Figs. 2 and 3 compare our findings for three sets of data: IDSP Gujarat, data for Gujarat in Annual NHP of CBHI and data for the rest of India in Annual NHP of CBHI from 2005 to 2011 for average reported cases and annual trends. We juxtaposed the rates from these data sets against rates available in the literature.

**Table 2:** Comparative case rates for selected diseases as reported by IDSP Gujarat, NHP for Gujarat, NHP for India and literature

Diseases	Average annual reported cases by IDSP Gujarat (2005–2011)	Average annual reported cases in NHP of CBHI for Gujarat (2005–2011)	Average annual reported cases in NHP of CBHI for India (2005–2011)	Trendlines of annual reported total cases by IDSP Gujarat, NHP of CBHI for Gujarat, NHP of CBHI for India	Calculated case rates from number of cases as indicated in literature with their settings and period.
Enteric fever	26.71	12.64	80.57	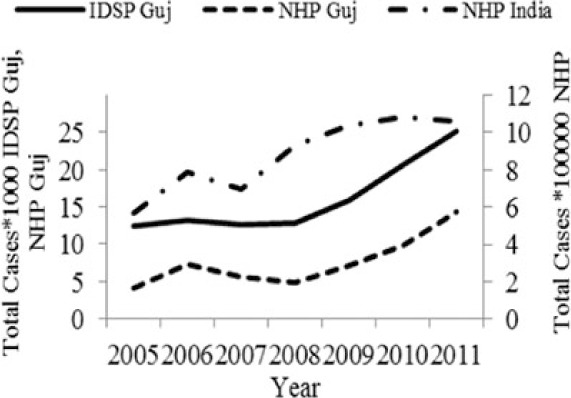	493.5/100,000 person years in two urban slums of Kolkata during Nov 1, 2003–Oct 30, 2004 (21).
Viral Hepatitis	24.76	8.53	10.85	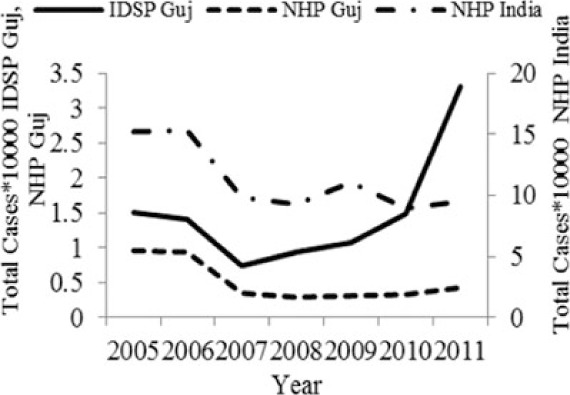	1) 4.5–100% Seroprevalenceof HAV antibodies in children and adults (22). 2) 3.2% HAV and 8.6% HEV antibody prevalence in school children from Chennai in 2002 (23).
Cholera	0.40	0.19	0.25	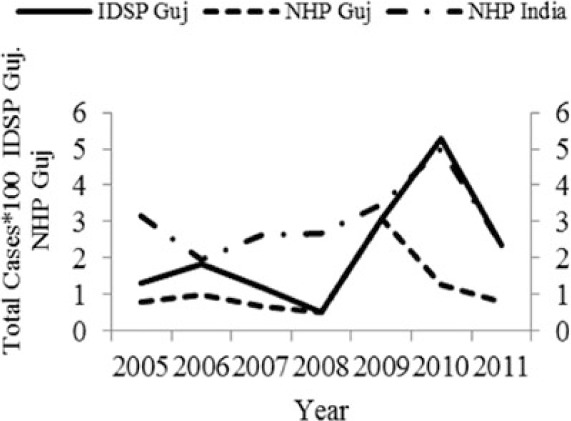	1) 1.3 incidence rate/1,000/year in urban slums in Kolkata during 2007–2010 (24). 2) Annual incidence rates 1.64/1000 population Kolkata during 2003–2005 (25).
Dengue	2.46	2.21	1.27	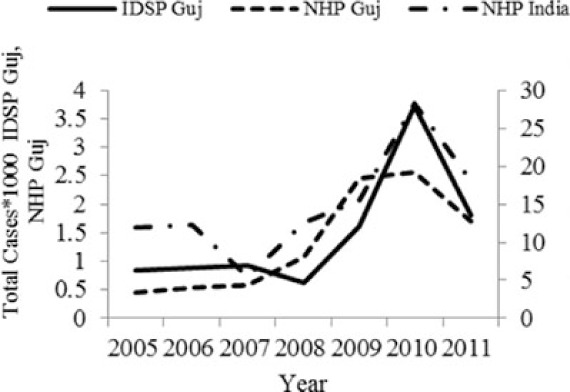	0.21/100,000 population, 2010 NVBDCP data for Gujarat (3).
Chikungunya	0.51	0.30	0.35	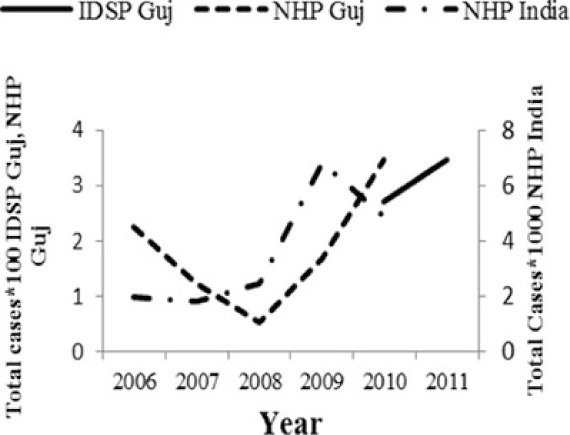	150/100,000 population (26).
Measles	1.67	0.74	2.80	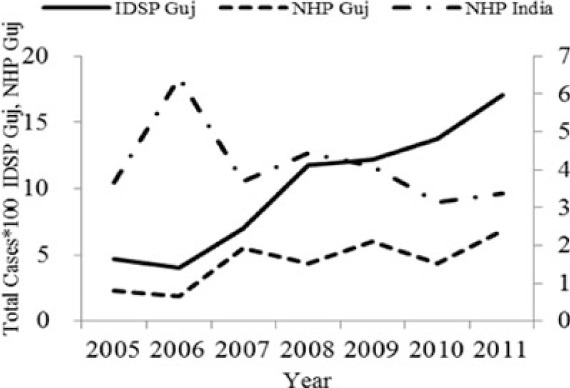	1) 2.42 per/100,000 population WHO observatory data as of 2 Oct 2012 (27). 2) 11.20% Measles in Ahmedabad slums in February 2000 (28).
Diphtheria	0.35	0.22	0.35	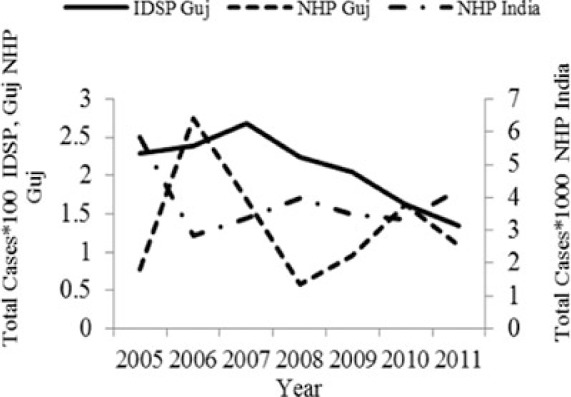	0.29/100,000 population WHO observatory data as of 2 Oct 2012 (27).

Figure 2 shows that compared to the average annual cases reported from the rest of India by CBHI over the 7 years, IDSP Gujarat reported approximately double of this average for four diseases, namely viral hepatitis, cholera, dengue and chikungunya; while it reported approximately half and one-third of the national average cases for measles and enteric fever, respectively.

The annual reported cases in NHP of CBHI for Gujarat (Column 3, Table 2) may be considered indicative of the efficiency of 1) the reporting between the state health department and national CBHI apparatuses; and 2) the expression of this reporting in the NHP. Trend lines comparing enteric fever rates in these two sets of data show a very similar progression of slopes, though the NHP reports only half the cases that the IDSP does throughout the 7 years (Column 3, Table 2). Trend lines for viral hepatitis and measles drop off after 2007 and for cholera after 2009 indicating decreasing reporting of these diseases.

Comparative trend lines between all three sets of data show a good correlation only for dengue. For all other diseases, the national and state trends have been quite different from each other.

Compared to average annual cases reported by IDSP Gujarat, the NHP for Gujarat reported half to one-third of this average for all the selected diseases (Fig. 3).

## Discussion

IDSP Gujarat, since it began in 2004, has been reporting an increasing trend of six of our seven selected diseases, excluding diphtheria. Thus, it appears that a state level monitoring system such as the IDSP holds more promise as a surveillance tool since it has shown a steady rise in reporting of cases. However, the NHP Gujarat has reported less than half the cases reported by the IDSP Gujarat. In an ideal data transmitting system, the two data sets would have shown similar case rates. Our comparative exercise has also revealed that two of the water-borne diseases, enteric fever and viral hepatitis cause the highest burden in Gujarat, while the vector-borne and vaccine-preventable groups exhibit much smaller numbers.

Water-borne infections: Enteric fever, viral hepatitis and cholera show variable reporting characteristics. Enteric fever, as per Gujarat IDSP, is being detected in rural areas and causes the highest burden of cases compared to the other selected diseases. However, this rate is actually one-third the national average reported by the NHP, indicating that Gujarat is actually reporting much less number of cases of enteric fever than the rest of the country. In contrast, viral hepatitis rates indicate that the state is reporting double the national average. Cholera cases, though reported in very small numbers by Gujarat, are double the national average and also exhibit the highest urban proportion of all the diseases.

This diversity of the case rates of these three water-borne diseases from the national average and their variable rural–urban characteristics is difficult to explain. The inconsistent data collecting and reporting efficiencies, private sector proportions, types and quality of water supply systems across the state and the country could be responsible for an immense range of contributory factors. That the IDSP is reporting increasing numbers of all these cases may be seen as a reassuring sign of improving surveillance for these diseases in the state.

Comparison of national rates with academic literature for these three water-borne infections also reveals similar wide differences. Surveillance studies undertaken during 2003–2010 for the estimation of enteric fever and cholera have reported 6 and 10 times more lab-confirmed cases, respectively, than the national average reported by NHP between 2005 and 2011. According to IDSP Gujarat, the case rate for enteric fever in the state is only one-third of the national enteric fever rates, closer to the lower, more heartening rates in Chinese and Vietnamese endemic sites (21). National rates for cholera are a third to sixth of that in the literature (29). Kanungo et al. state that although India had reported cholera cases and deaths to WHO regularly from 1997 to 2006, their analysis of published papers on outbreaks during this period found gross underreporting of the number of cases (30). They attributed this mainly to an inadequacy of laboratory capacity and surveillance in peripheral healthcare centres.

Vector-borne infections: Dengue and chikungunya reporting also display diametric characteristics. While IDSP Gujarat reported double of the NHP national average for cases of dengue, it reported only one-tenth of the national average for cases of Chikungunya. The good correlation of the three data sets for dengue indicates that the reporting by NVBDCP Gujarat to the NHP agrees closely with that of IDSP Gujarat. Reporting for chikungunya exhibits an interesting feature—while IDSP Gujarat has reported extremely few cases in 2010–2011, the NVBDCP has been reporting cases to the NHP since 2005. Reporting by IDSP Gujarat seems to have begun after a seminal paper, which proposed that the chikungunya epidemic of August–September 2006 in Ahmedabad, the capital of Gujarat, was responsible for excess deaths in the city, was published (31). Estimated case rates in Gujarat in the epidemic year of 2006 were actually about half the average case rates across the 15 states that had been affected by the epidemic (26).

Vaccine-preventable infections: Measles and diphtheria rates reported by IDSP Gujarat are higher than those reported by NHP Gujarat indicating a better capturing of these two diseases. The rates reported by NHP for India were slightly different than that reported by the WHO Observatory for the same time span. But since the reported cases of these diseases are very small, this difference is probably not noteworthy. India contributed 8.5% of the global number of measles cases and 71.3% of the global number of diphtheria cases in 2011, topping the list of contributing countries for diphtheria (27).

The limitations of this study are first, IDSP estimates are conservative. The analysis presented above is based on data reported by the nascent IDSP system. Although the majority of providers currently do not report to it, more reporting units are being enrolled every year. Variations in laboratory-confirmed disease cases due to outbreaks have not been accounted for in our calculations. We also assumed a constant population over the study period for estimating the case rates. Second, in practice, larger hospitals and medical colleges generally undertake laboratory investigations of suspected cases of these diseases. Clinical diagnosis unsupported by laboratory investigations is routine in smaller and single healthcare provider practices and therefore information slips through the surveillance network. Private practitioners have significantly worse knowledge of correct diagnosis and treatment (32). Third, India has a multiplicity of treatment providers and medical systems. The distribution of these providers is skewed and we see a disproportionately higher number of allopathic providers in the urban areas who are more likely to use lab investigations than the non-allopathic providers in rural areas (33). Finally, we were unable to source annual IDSP data at the national level and for the remaining eight states where the project is presently functional.

Despite these limitations, this work, to our knowledge, is the first to document case rates at a state-level in India and recognise the contribution of the IDSP system for epidemiological considerations. Our analysis shows that the existing surveillance system in the state is predominantly reporting urban cases. Reports of enteric fever and measles are less than half of the national average, while cholera, viral hepatitis and dengue are nearly double.

The surveillance studies are focussed and conducted in endemic locations and therefore show high incidences of these diseases. Therefore, it is expected that their rates will be higher in the literature than the national average reported by the NHP of CBHI. However, for a large country such as India, such few and far between studies are not adequate in understanding the burden of these infectious diseases across the country.

Although infectious diseases such as TB and HIV have higher burdens than enteric fever and hepatitis, there is a need to also begin addressing these latter diseases with a systems approach. Generating reliable data on these diseases is the obvious first step. The IDSP is ideally placed to take on this role. This could be vastly advanced by a more robust data access policy of IDSP data such as creating a central IDSP data repository for all the nine IDSP states across the country along the lines of the WHO Global Laboratory Directory for Cholera and Global Health Observatory for vaccine-preventable diseases. An open access policy to IDSP data would encourage involvement from interested stakeholders, especially private practitioners and researchers. This would contribute to a robust data collection and collation system. The IDSP can potentially be strengthened into a sound surveillance and response mechanism capable of taking on the challenge of reversing the endemicity of these diseases.

## References

[1] Greenstone G (2009). A commentary on cholera: the scourge that never dies. BCMJ..

[2] De M (2005). Cholera: the scourge of India and Bangladesh. Curr Sci..

[3] Chakravarti A, Arora R, Luxemburger C (2012). Fifty years of dengue in India. Trans R Soc Trop Med Hyg..

[4] United Nations Human Settlement Programme (2003). The Challenge Of Slums: Global Report On Human Settlements 2003.

[5] Jones KE, Patel NG, Levy MA, Storeygard A, Balk D, Gittleman JL (2008). Global trends in emerging infectious diseases. Nature..

[6] Shope R (1991). Global climate change and infectious diseases. Environ Health Perspect..

[7] Project Implementation Plan (2004). Integrated Disease Surveillance Project.

[8] The World Bank Project Information Document (PID) Report No. PID10512 (2001). Document of The World Bank. http://www-wds.worldbank.org/external/default/WDSContentServer/WDSP/IB/2001/07/14/000094946_01071304251057/Rendered/PDF/multi0page.pdf.

[9] The World Bank Updated Project Information Document (PID) Report No AB52 (2003). Document of The World Bank. http://www-wds.worldbank.org/external/default/WDSContentServer/WDSP/IB/2003/05/23/000094946_03050704115685/Rendered/PDF/multi0page.pdf.

[10] The World Bank Restructuring Paper on Proposed Project Restructuring of Integrated Disease Surveillance Project to the Republic of India (2010). http://www-wds.worldbank.org/external/default/WDSContentServer/WDSP/IB/2012/04/03/000333038_20120403014358/Rendered/PDF/677970PJPR0v100rch03000201200final.pdf.

[11] Ministry of Health & Family Welfare (2009). IDSP Training Manual for Paramedical Staff for Hospital Based Disease Surveillance.

[12] State Surveillance Unit Gujarat, Integrated Diseases Surveillance Project (2011). Integrated Diseases Surveillance Project Annual Report 2011.

[13] Ministry of Health and Family Welfare. National Health Profile (2011). Central Bureau of Health Intelligence, Government of India. http://www.cbhidghs.nic.in.

[14] Strategic Information Management Unit, National AIDS Control Organisation (2010). United Nations General Assembly Special Session Country Progress Report.

[15] Central TB Division, Ministry of Health and Family Welfare (2012). Revised National TB Control Programme Annual Status Report, TB India.

[16] World Health Organization, Rotary International, Centre for Disease Control, UNICEF (2012). Polio Global Eradication Initiative Report, Polio in India: Fact Sheet.

[17] Ministry of Health & Family Welfare (2005). Surveillance of Acute Flaccid Paralysis Field Guide.

[18] Ministry of Health and Family Welfare (2007). Strategic Action Plan for Malaria Control in India 2007–2012.

[19] Singh N (2009). A new global malaria eradication strategy: implications for malaria research from an Indian perspective. Trans R Soc Trop Med Hyg..

[20] Sharma V (1999). Current scenario of malaria in India. Parassitologia..

[21] Ochiai RL, Acosta CJ, Danovaro-Holliday MC, Baiqing D, Bhattacharya SK, Agtini MD (2008). A study of typhoid fever in five Asian countries: disease burden and implications for controls. Bull World Health Organ.

[22] Mathur P, Arora N (2008). Epidemiological transition of hepatitis A in India: issues for vaccination in developing countries. Indian J Med Res..

[23] Mohanavalli B, Dhevahi E, Menon T, Malathi S, Thyagarajan SP (2003). Prevalence of antibodies to hepatitis A and hepatitis E virus in urban school children in Chennai. Indian Pediatr..

[24] Kanungo S, Sur D, Ali M, You YA, Pal D, Manna B (2012). Clinical, epidemiological, and spatial characteristics of Vibrio parahaemolyticus diarrhea and cholera in the urban slums of Kolkata, India. BMC Public Health..

[25] Ali M, Lopez AL, You YA, Kim YE, Sah B, Maskery B (2012). The global burden of cholera. Bull World Health Organ.

[26] Krishnamoorthy K, Harichandrakumar KT, Krishna AK, Das LK (2009). Burden of Chikungunya in India: estimates of disability adjusted life years (DALY) lost in 2006 epidemic. J Vector Borne Dis..

[27] World Health Organization (2012). Global health observatory.

[28] Bhagyalaxmi A, Kedia G, Rawal GS (2007). Study of incidence of measles and vaccination coverage in Ahmedabad urban slums. Indian J Public Health..

[29] Sur D, Deen JL, Manna B, Niyogi SK, Deb AK, Kanungo S (2005). The burden of cholera in the slums of Kolkata, India: data from a prospective, community based study. Arch Dis Child..

[30] Kanungo S, Sah BK, Lopez AL, Sung JS, Paisley AM, Sur D (2010). Cholera in India: an analysis of reports, 1997–2006. Bull World Health Organ..

[31] Mavalankar D, Shastri P, Bandyopadhyay T, Parmar J, Ramani KV (2008). Increased mortality rate associated with chikungunya epidemic, Ahmedabad, India. Emerg Infect Dis..

[32] Basu S, Andrews J, Kishore S, Punjabi R, Stuckler D (2012). Comparative performance of private and public healthcare systems in low-and middle-income countries: a systematic review. PLoS Med..

[33] Ministry of Health & Family Welfare (2005). Report of the National Commission on Macroeconomics and Health.

